# Including the child’s voice in research from a longitudinal birth cohort: insights from the ROLO young person’s advisory group

**DOI:** 10.1186/s40900-023-00411-y

**Published:** 2023-02-09

**Authors:** Anna Delahunt, Sophie Callanan, Sarah Louise Killeen, Ciara M. McDonnell, Fionnuala M. McAuliffe

**Affiliations:** 1 UCD Perinatal Research Centre, School of Medicine, The National Maternity Hospital, University College Dublin, Dublin 2, Ireland; 2 Department of Paediatric Endocrinology and Diabetes, Children’s Health Ireland, Temple Street and Tallaght, Dublin, Ireland

**Keywords:** Children, Public and patient involvement, Birth cohort, Research engagement, Young Person’s Advisory Group

## Abstract

**Background:**

Public and patient involvement (PPI) through Young Person’s Advisory Groups (YPAG) enables children to provide guidance and insight into research activities. PPI is an important characteristic of research, however, to date, most collaboration has been with adults. Also, few YPAGs have been established within the Irish setting. The ROLO (Randomised cOntrol trial of a LOw glycaemic index diet in pregnancy to prevent macrosomia) YPAG was established in July 2020 to identify the research priorities of a group of healthy Irish children who are part of a longitudinal birth cohort. We aimed to describe this process and the key insights to date.

**Methods:**

The ROLO study is a longitudinal birth cohort which has followed-up mother–child dyads at multiple timepoints over 10 years. Mothers actively involved in the study were contacted by the research team to invite their ROLO child and older sibling to participate in the YPAG. Meetings were conducted virtually between July 2020 and February 2022. Researchers encouraged free expression of views amongst the children regarding their research interests. Meetings were recorded, transcribed verbatim and analysed for themes based on the topics most frequently discussed and considered important to participants.

**Results:**

In all, seven ROLO children and six older siblings attended four ROLO YPAG meetings. Participants were aged between nine to fifteen years old. Four key themes were identified; study children viewed their identity as part of a longitudinal birth cohort as positive and unique; study children considered the fitness test and body measurements as fun aspects related to their participation; all children considered the impact and use of social media as an important form of communication; and all participants expressed interest in attaining new health-related information and learning opportunities. Children suggested topics such as mental health, future viruses, organ transplants, cancer, and the effect of technology and chemicals on the body were important for future research.

**Conclusion:**

The ROLO YPAG offers promising scope for continued collaboration. The themes identified from the meetings contribute to a gap in the literature which will guide future research activities, particularly with children, in view of study design, relevance, and by communication strategies.

*Trial Details*: ISRCTN54392969 registered at www.isrctn.com.

**Supplementary Information:**

The online version contains supplementary material available at 10.1186/s40900-023-00411-y.

## Plain English summary

The ROLO pregnancy study took place in the National Maternity Hospital in Dublin Ireland. It started in 2007 and ended in 2011. The researchers recorded what women were eating. They also measured the weight of the baby at birth. Since then, ROLO mothers and their children were invited to come back to the study. Now the children of the study are 9–11 years of age.

The researchers invited members of the ROLO study to speak with them. They wanted to know what research was important to them. They set up a group called the ROLO Family Advisory Committee in 2017. This group of parents and researchers meet once a year. The group thought it was important to include children as well. They made a new group called the ROLO Young Person’s Advisory Group in 2020. The group has 7 ROLO children and 6 older siblings. The members are aged between 9 and 15-years-old. The children and researchers have met four times so far.

The researchers found four key themes. Study children saw their identity as being part of a longitudinal birth cohort as positive and unique. Study children liked the fitness test and body measurements. All children thought that social media was an important form of communication. All children were interested in learning new information on how their bodies worked.

Involving this group of children is important. It will make our research more relevant. Other researchers who want to involve children can learn from our experience.

**Supplementary Information:**

The online version contains supplementary material available at 10.1186/s40900-023-00411-y.

## Introduction

In recent times, greater attention has been directed towards the importance of the voice of children in matters that affect them [1]. As outlined by the United Nations Convention on the Rights of the Child (UNCRC) 1989, children are entitled to freedom to express their views and for them to be given due weight [1]. Although this global shift in thinking has led to significant changes, opportunities for the voice of children to be heard and acted upon remain limited [2]. Adults tend to interpret and speak on behalf of young people. Children are often subjected to external influence, and their own experiences can be disregarded [2, 3]. The diverse perspectives of children are valuable to society, as they may propose new questions and innovative ideas that adults do not perceive [3].

One fundamental subject affecting all young people is paediatric health research [4]. All children of society have a pivotal role in the improvement of health outcomes, by sharing their views regarding research significance and relevance [5, 6]. The importance of involving children in health research is endorsed by several international health bodies, while the UNCRC states that young people should be involved in any research that impacts them [1, 7, 8]. To facilitate this role, public and patient involvement (PPI) serves as a suitable platform for young people to guide and direct research that is most representative of their needs [3, 4].

Efforts to include PPI is an important aspect of paediatric health research that is increasingly delivered through models of consultation, user-led research, and collaboration [6]. Collaboration with Young Person’s Advisory Groups (YPAGs) is one model that empowers children by involving them in decision making and action planning, while increasing confidence and research skills [3, 4, 6]. This can improve recruitment and retention rates, guide the acceptability and feasibility of studies, and increase research relevance [3, 4, 9].

The ROLO (Randomised cOntrol trial of a LOw glycaemic index diet in pregnancy to prevent macrosomia) study was a randomised controlled trial of a low glycaemic index diet during pregnancy versus routine antenatal care. The aim was to reduce the recurrence of macrosomia in secundigravida women attending the National Maternity Hospital, Dublin, Ireland (2007–2011) [10]. It has developed into a well-recognised longitudinal birth cohort study, with follow-up of 759 mother–child dyads at multiple timepoints in childhood [10–15].

The ROLO Family Advisory Committee was established in 2017. This group of parents from the original ROLO study expressed interest in meeting annually to identify research priorities and provide their opinions on research [16]. This partnership has enabled mutual learning opportunities, successful grant funding applications, and a recent collaborative publication between researchers and parents on the role of PPI in the ROLO study [16]. Discussion with the group raised the importance of also listening to the child’s views about our research and proposed the establishment of a PPI group for the ROLO children [16]. It is important to acknowledge the needs of PPI participants and researchers are obliged to take heed in future practice [4]. Practical resources and guidance for involving children in research have been published, however, the formation of YPAGs in health research in Ireland is lacking [17]. Despite the challenges of the COVID-19 pandemic, researchers recognised the importance of this gap that had been identified and formed the ROLO YPAG in July 2020.

The ROLO YPAG is a unique group of healthy Irish children between the ages of 9 to 15 years old who can provide views and opinions related to paediatric health research. This report aims to share our experiences of developing the ROLO YPAG and provide insight into the main themes identified.

## Methods

### The ROLO family advisory committee

To incorporate PPI into the ROLO study, the ROLO Family Advisory Committee was established in 2017. All women who were still active participants from the primary ROLO pregnancy study as of October 2017 (n = 650), along with other parents and guardians of the ROLO children, were contacted and invited to participate. The process of establishing the committee, including details on the recruitment of members, communication strategies, and structure of the meetings have been described previously [16]. In brief, the committee has met five times over the past five years, with the research team (2018 and 2022). The initial group, (2018), consisted of 16 ROLO mothers and one father. In April 2022, a total of 20 ROLO mothers and 2 fathers expressed interest in being actively involved. The first two meetings between the research team and the committee were held face-to-face in the National Maternity Hospital, whilst the following three meetings were held virtually due to COVID-19 pandemic restrictions. During these meetings, parents were encouraged to share their opinions about current research projects, and potential future projects, followed by an open discussion about relevant health questions [16].

### Establishing the ROLO young person’s advisory group

While clear guidance has been issued in the UK that ethical approval is not a formal requirement to conduct PPI activities with children, similar direction has not yet been devised in Ireland [18, 19]. Therefore, ethical approval was sought to establish this group to guard the participation of attendees. An ethical amendment to the ROLO Preteen study to establish YPAG was sought in April 2020 from the UCD Research Ethics Committee and ethical approval was granted in May 2020 (LS-15-06-Geraghty-McAuliffe). The aim establishing the ROLO YPAG is to understand the key outcomes of importance for children relating to the health of themselves and their families, and to seek advice related to how the research agenda can be more relevant to the child’s needs.

### Recruitment of the ROLO young person’s advisory group

The ROLO YPAG is a parent-selected group of children who are involved in the ROLO longitudinal birth cohort and their older siblings. All mothers who were still active in the ROLO longitudinal birth cohort (n = 625) were contacted by email by the research team and informed about our plans to set up a YPAG. It was communicated in this email that their ROLO study child and their older sibling were invited to participate. The purpose of inviting the older sibling to attend was to provide an external perspective within the group. Parents were encouraged to discuss the details of the group with their children to see if they would like to take part. Parents were asked to confirm their children’s interest in attending the next meeting by emailing or phoning the research team. Subsequently, parents whose children expressed interest in participating in the YPAG were invited to complete a short online survey created by the research team through the web-application SurveyMonkey (Momentive™ Inc., California, United States of America). The 5-item survey consisted of multiple choice and open-ended questions that were emailed to the parents. This collected information on their children’s availability and preferences for the size of the group that their children would feel most comfortable with. Most parents indicated a preference for the meetings to take place in small groups of no more than six to ten children. It was thought that this would facilitate a comfortable environment for the children. Prior to attendance of each meeting, informed consent by email was obtained from mothers to confirm their children’s involvement and to allow recording of the meeting. Verbal consent to participate and be recorded was then obtained from children at the start of every meeting. Terms of reference were developed to describe the purpose and structure of the YPAG.

### Communicating the purpose of the ROLO young person’s advisory group to children

To inform interested participants, child focused written information was developed to help children and their parents to understand the purpose of the YPAG and what to expect at YPAG meetings. This was emailed to the children’s mother (Additional file 1). Following the third YPAG meeting, researchers queried whether some of the children who had attended had a thorough understanding of the purpose of the YPAG or their role in it. Hosting meetings virtually thus far provided a barrier in ensuring full understanding. To help increase understanding of the group’s role, a short video explaining the role of the YPAG was designed by the research team with the children as the target audience. This was emailed to the mothers of children approximately two weeks before the fourth meeting. It was hoped that by using a more interactive form of communication that the key messages of the group's role would be better understood.

### Virtual meetings of the ROLO young person’s advisory group

The ROLO YPAG was established in July 2020 and has met four times with the research team between July 2020 and February 2022. Originally, meetings were planned to be held in person as a workshop style meeting but because of COVID-19 restrictions and protocols, all meetings have been held virtually thus far. The online telecommunications platform Zoom™ (Zoom™ Video Communications Inc., California, United States of America) was used for all four meetings [20]. This was considered the most appropriate interface for ensuring participant safety and confidentiality due to the increasingly improved security measures and our access to an advanced security account through our university affiliation. The Zoom™ platform was used to record the videotaped meeting so that the content of the meeting could be transcribed verbatim. Recordings were available for 30 days following the meeting before being automatically deleted. Each meeting lasted approximately one hour in duration. This length of time was considered appropriate to maintain interest.

### Content and delivery of the ROLO young person’s advisory group meetings

To devise the content and delivery of the meetings, the researchers relied on information from attending workshops hosted by the PPI Ignite Network at Dublin City University, speaking to colleagues who had previously engaged in PPI activities, experience from the ROLO Family Advisory Committee, and reading literature from those who had previously engaged with a YPAG [16, 17, 21–23]. The key focus of the initial meetings of the YPAG was to create familiarity between the children and the researchers, an important aspect of laying the foundations for a newly established YPAG [22]. This process was made easier by having small groups at each meeting. In addition, all study children had recently attended their ROLO Preteen follow-up visit. Researchers began each meeting with a short overview of the ROLO study to help put the children’s involvement in the YPAG into context. The use of icebreakers, interactive activities, whiteboards, polls, and quizzes were also included for 5 to 10 min to help to encourage discussion and put children at ease. Researchers aimed to guide, rather than direct, children through each meeting with several general topics being prompted by the researchers. These included what their experience was of being part of the ROLO study; physical activity; nutrition; health; social media; research and science. Researchers encouraged free expression of views and opinions regarding health-related research priorities and topics of importance, to enable themes and subthemes to naturally develop.

### Identification of themes from the ROLO young person’s advisory group meetings

Transcripts from the four meetings of the ROLO YPAG were analysed using thematic analysis, as per guidelines published by Braun and Clarke [24]. After the four meetings, two researchers (SC and AD) implemented an inductive, structured, and thorough coding and theme development procedure for each transcript to minimise potential bias. The identified themes and subthemes were independent of each other and reflected reoccurring patterns which were supported by quotations from the pseudo-anonymised transcripts. Triangulation was performed by a third, independent researcher (SLK). Discussion amongst the researchers (SC, AD, SLK) clarified the four most frequent and representative themes and subthemes.

### Engagement and retention strategies for the ROLO young person’s advisory group

The ROLO study has a strong history of participant engagement and retention, attributed to several communication strategies which have been described previously [25]. All mothers of the ROLO study were contacted to provide their children with the opportunity to take part in the YPAG, regardless of their attendance to previous ROLO study follow-ups. Efforts were taken to contact all participants including dissemination about what the group entailed through the use of posts on the dedicated ROLO Facebook page and providing information about the YPAG in the quarterly ROLO study newsletter. Annual handwritten birthday cards are also sent to each ROLO child, which includes a letter to encourage participants to update their contact details with the research team if necessary [25]. The same recruitment process was repeated before each YPAG meeting to allow new members to join. After the first three meetings, all participants received written recognition by email from the researchers to acknowledge their contribution. Researchers had discussed providing a token of appreciation at the outset of establishing the YPAG. However, this was revisited before the fourth meeting when the importance of providing an incentive became more apparent to researchers as their experience further developed from attending workshops and learning from other groups. Therefore, children who attended the fourth meeting received a certificate of attendance and a small token of appreciation for their participation. Six of the seven children that attended the fourth meeting had attended one of the previous three meetings, and therefore the researchers acknowledged that it was an important way to retain the group's engagement and to show recognition for their input to date.

## Results

### Description of participants at the ROLO young person’s advisory group meetings

In all, 7 ROLO children and 6 older siblings have taken part in the YPAG to date. Attendees consisted of 6 sibling pairs and one study child who participated without their older sibling. Participants ages ranged from 9 to 15-years-old with 3 sibling pairs attending more than one meeting, and 3 sibling pairs and one study child attending at least one meeting. The first three meetings consisted of smaller groups of attendees (n = 4–6), while the most recent meeting in February 2022 had the highest number of participants (n = 7). Of the 7 ROLO study children who have participated to date, 5 were from the intervention group in the primary study. All study children involved in the YPAG had attended a ROLO follow-up study visit at 9 to 11-years.

### Themes identified from the ROLO young person’s advisory group meetings

**Fig. 1:**
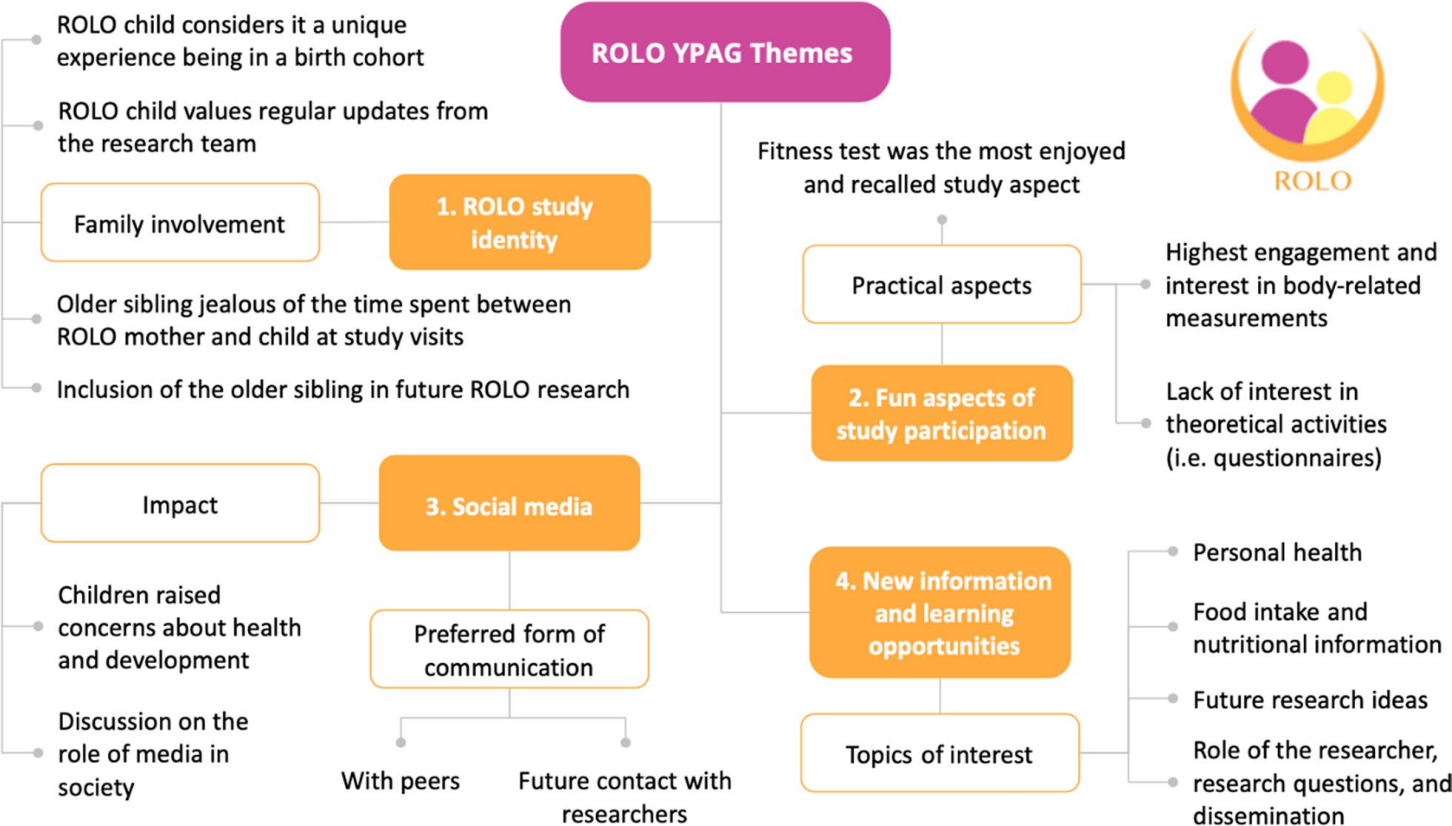
Themes and subthemes identified from the ROLO Young Person’s Advisory Group meetings. *ROLO* Randomised cOntrol trial of a LOw glycaemic index diet in pregnancy to prevent macrosomia, *YPAG* Young Person’s Advisory Group

The four main themes identified from the YPAGs are displayed in Fig. 1. They have been identified as: ROLO study identity, fun aspects of study participation, social media, and new information and learning opportunities.

#### Theme 1: ROLO study identity

This theme relates to what it means for the study children to be part of a longitudinal birth cohort. Researchers received positive insights from the study children regarding their experience of being part of a longitudinal birth cohort. It was identified that study children indicated that their involvement from birth is a unique opportunity compared to their peers and siblings.

“Usually, kids wouldn’t get to know that stuff about themselves.” (Study child 1, meeting 2, aged 13 years)

In discussions, study children valued the recognition of their participation through receiving annual handwritten birthday cards and quarterly study newsletters. Study children looked forward to receiving them and advised researchers to continue sending them through the post so that they can receive a physical copy. However, some were unsure of their connection to the research team, highlighting a potential gap for improved future communication and engagement.

“Oh, these are the people that send them?” (Study child 2, meeting 1, aged 11 years)

Frequent discussion emerged amongst the YPAG regarding the participation of older siblings in the ROLO study. Children shared their awareness of the prominent differences between siblings, by comparing their individual interests, behaviours, and physical appearance. Feelings of jealously were also raised by older siblings related to the study child spending one-to-one time with their mother at their ROLO study visit.

“I don’t really know, but I’d say I might have been a bit jealous. I think it’s because my mom was bringing (ROLO child) or something like that. I felt a bit jealous that (ROLO child) got more time with mum.” (Older sibling 1, meeting 2, aged 11 years)

On several occasions, the YPAG proposed that the inclusion of siblings in the ROLO study warrants future exploration.

“I think the differences between brothers and sisters will be kind of cool.” (Older sibling 2, meeting 2, aged 14 years)

#### Theme 2: fun aspects of study participation

This theme relates to what the fun aspects are of being part of the ROLO study. Discussion amongst the YPAG highlighted fun aspects of the study participation that were largely linked to physical activities. Study children viewed their experience of attending ROLO study visits positively and expressed that this was related to fun. Similarly, the YPAG commented that the fitness test was a source of enjoyment and a preferred measure of testing physical fitness for the purpose of research. The fitness test was also repeatedly recalled by multiple study children as the most favoured measurement from the ROLO study visit, while the blood test was viewed negatively by most study children.

“And well, I remember from my last thing we did like the bleep test and I found that kind of fun” (Study child 1, meeting 2, aged 13 years)

Study children frequently recalled physical measures from their study visit such as blood pressure, the fitness test, the dual-energy absorptiometry scan, and the blood test. Interestingly, these measurements all share the common characteristic of being related to the body, which involve active participation and interaction for the child at the visit. This contrasts to a lack of interest related to the theoretical aspects of the study such as participant questionnaires that children did not recall or refer to during the YPAG meetings.

“I got blood taken… And then there was a bleep test” (Study child 3, meeting 4, aged 10 years)

#### Theme 3: social media

This theme relates to the importance and usage of social media in this age group. The researchers prompted discussion about social media as a potential topic of interest. The YPAG agreed that social media engagement is the main form of communication with their peers. Children considered it part of their normal daily routine which they enjoyed. The YPAG suggested that social media may be an appropriate and highly effective mode of contact between researchers and study participants regarding participation in future study activities.

“Em yes, it’s the only way I contact my friends” (Older sibling 3, meeting 1, aged 13 years)

When engaged in discussion, children shared the platforms that they enjoyed the most, which provide insight into the trending nature of social media in this age group.

“I despise TikTok, and my parents haven't allowed me to get Instagram so I only use snapchat really” (Older sibling 3, meeting 2, aged 13 years)

The YPAG also showed significant interest in the power of social media and the influence of technology. This was evident at multiple timepoints as children availed of opportunities across various discussions to raise questions regarding their potential impacts on both their health and the wider online platform. This highlights a common awareness amongst the group of the possible unknown dangers linked with media usage over time.

“Can social media take over the Internet? Has it already taken over? Who runs social media?” (Study child 3, meeting 3, aged 9 years)

#### Theme 4: new information and learning opportunities

This theme relates to children expressing a desire in seeking new information and learning opportunities about; themselves, health, nutrition, and the research process. Discussion amongst the YPAG raised the idea that their involvement in the ROLO study was considered a gateway to learn more about themselves and their health.

“Well, it’s, it’s kind of like fun to like find out stuff about yourself that you didn’t really know” (Study child 1, meeting 2, aged 13 years)

The YPAG also significantly engaged in discussion when posed with the question regarding their future research topics of priority. Children advised researchers to explore a range of innovative issues such as addiction, mental health, future viruses, organ transplants including heart and bone health, intellectual disabilities including dyslexia and autism, exercise, cancer, genetic testing, environmental issues, and the effect of technology and chemicals on the body. The researchers noted that children did not require further prompts relating to this question and were eager to suggest several issues, calling out multiple ideas consecutively. The reoccurring topics that were raised across meetings included viruses, genetic testing, and factors impacting development. These ideas provide novel perspectives for future research questions in the ROLO study and reflect the research priorities of young people in society.

“Bone transplants, mental health, autism, dyslexia” (Study child 3, meeting 4, aged 10 years)

The YPAG also expressed notable curiosity related to nutrition and food. Children highlighted their doubts regarding the nutritional value of food groups, the positioning of food groups on the food pyramid, and the link between diet and disease risk. The YPAG suggested that nutritional information displayed on a food pyramid could be delivered in a clearer format, such as using physical food as a visual guide.

“Can you get a disease by eating something?” (Study child 3, meeting 3, aged 9 years)

Similarly, the research process sparked interest amongst the YPAG. Children queried the role of the researcher, formulation of research questions, and the dissemination of research results. In addition, children were prompted by the researchers to share their views on what research means to them, why research is important, and what skills are necessary for carrying out research.

“Helps you figure out like maybe; I have been studying it. You know. I know, I mean like, and say like, if you want to figure out your family tree, huge huge huge pieces of your DNA” (Older sibling 1, meeting 4, aged 12 years)

## Discussion

The ROLO YPAG was recently established with the aim of identifying the research priorities of a group of healthy Irish children born into a longitudinal birth cohort study. This study provided an opportunity to explore key themes that were most frequently discussed and considered of importance to the YPAG which include; ROLO study identity, fun aspects of study participation, social media, and new information and learning opportunities. Throughout discussions related to these themes, the YPAG provided useful suggestions for future research activities. The anticipated benefits of this partnership between children and researchers places the inclusion of PPI as a fundamental aspect of the ROLO study.

The success of longitudinal birth cohorts are critically determined by sustained relationships between researchers and participants for many years [26]. The YPAG indicated that strengthening children’s identity in a birth cohort may support their partnership with researchers. The concept of being involved in longitudinal research from before birth may be challenging and barriers to greater participation arise as cohort members age [26]. Regular engagement and consultation is considered a high priority in studies of longitudinal design to support sustained retention and participation [26]. In 2013, Lucas et al. [26] evaluated the strategies used amongst eighty-four European birth cohorts to support wider engagement with young people. Many reported the use of websites, newsletters, birthday cards, and social media, which are also implemented in the ROLO study [25, 26]. Despite this, just two cohorts reported actively and regularly consulting with young people [26]. This research highlights a current gap in consultation with children regarding their experience in a longitudinal study, which the ROLO YPAG aimed to address. Thus, increased efforts to consult with young people involved in studies of longitudinal design may reinforce their value of being affiliated with research.

The acceptability of measurements is a fundamental aspect of longitudinal research to support long-term retention [26]. Data collection methods which are disliked by participants are more likely to be left incomplete or inaccurate [26]. Our YPAG advised that they preferred the physical and interactive elements of the research activities, rather than theoretical tasks that resemble the schooling environment. Recognising this will inform our future research planning so that practical research methodology is included, and this may help to maintain long-term engagement. Similar guidance was provided from 7-year-olds involved in a longitudinal clinical birth cohort in Finland [27]. Children considered the most popular tests from their study visits as those that mimicked play including moving, walking, running or playing with a computer [27]. Incorporating this information into future study planning may encourage sustained involvement.

Online communication platforms such as social media are important to young people [28]. Children in the ROLO YPAG are in the early years of adolescence, which coincides with increased demand for autonomy and usage of technology [28]. The YPAG recommended that social media may be an appealing mode of contact between researchers and study participants. It is well-recognised that researchers should tailor the involvement of young people in research to suit their personal lifestyles, abilities, and interests [29]. Hence, supporting the autonomy of young people by using a variety of secure online platforms for the purposes of recruitment, consent, and research activities may interest adolescent ages [30]. Online communication between researchers and young people is one example of successful collaboration by the Oxford Neuroscience, Ethics and Society Young People’s Advisory Group (NeurOx YPAG) [31]. Young people aged 14–18 years are recruited to the NeurOx YPAG through an online form and members contribute to an online blog after each meeting to share their YPAG experiences [31]. Additionally, the NeurOx YPAG recently advised researchers to collaborate with social media influencers for the purpose of youth-led dissemination of results [32]. Other international YPAGs have incorporated the use of WhatsApp groups to communicate and engage with adolescent participants [33]. These examples may guide future communication strategies in health research with adolescent ages.

The ROLO YPAG advised researchers to explore a range of innovative ideas that reflected their future research topics of interest. A previous example of how PPI has contributed to the research agenda of the ROLO study was in relation to the topic of food fussiness/selective eating. Parents from the ROLO Family Advisory Committee identified this topic as an important area of research [16]. This feedback prompted the submission of a research proposal on this topic, which consequently resulted in a successful grant application for a PhD post to research this topic [34–36]. The research team anticipate similar action can be achieved with the suggestions from the ROLO YPAG. Our understanding that children are keen to learn about health and research correlates with advice from previous YPAG collaborations. In 2021, researchers reflected on their delivery of a YPAG in palliative care and health research at a Canadian secondary school [37]. Roach et al. [37] concluded that the 10- to 13-year-olds involved in this group advised that they are interested in learning about research methods, devising their own research, and being exposed to new topics. This raises the importance that research findings should be easily accessible for young people, which respects their role in the research process [29, 38]. As a result, our research in the ROLO study would benefit from increased focus towards child-friendly dissemination. Interactive models, diagrams, videos, and infographics have all been noted as effective forms of communicating complex research messages to children [38].

Several practical insights gained by researchers from the ROLO YPAG can be used to guide the next direction of this group and future YPAGs. Effective recruitment strategies were considered essential for the successful formation of the YPAG. Researchers wanted to acknowledge the busy lifestyles of participants, thus offering a range of dates and times during school holidays was considered the most suitable approach. Significant focus towards the preparation of appropriate and engaging material for meetings was deemed worthwhile. The use of whiteboards, polls, and quizzes, while avoiding overreliance on PowerPoint slides encouraged discussion and created a relaxed environment. Researchers ensured the content of the meetings were kept to a minimal, and pictures and colour were used to avoid overloading information. In the future, in-person meetings, will help to mitigate some issues around flow of conversation. This will also allow increased interactive content. Pitching the subject matter of the meetings so that it is at an appropriate level for both preteen and teenage children presented a challenge and is an area that researchers are aware requires further thought. As the group developed, researchers recognised the importance of acknowledging participants input. After the fourth meeting, each child received a small book token, certificate of attendance, and a thank you letter as an expression of appreciation from the research team. On reflection, the researchers acknowledge that it would have been fairer practice to introduce the token of appreciation from the outset for the purpose of consistency and to ensure all children feel their time and contribution to the group is valued.

To gain practical insights from the children, participants were given a short opportunity at the end of each meeting to provide verbal feedback on their experience of contributing. Researchers prompted children to reflect on whether they enjoyed the content and structure of meetings thus far. Researchers asked participants if they would be interested in various future activities related to the group such as attending in-person meetings, designing a logo for the ROLO YPAG, along with reviewing information and consent forms for the ROLO study. Researchers received positive gestures in response from children including thumbs up and nods of approval, indicating that participants are interested in taking on these roles within the group. Children were also given the opportunity to suggest changes to the format of the meetings. As the YPAG evolves, researchers aim to place greater emphasis on gaining insights and feedback from children.

The learning experiences gained thus far by establishing the ROLO YPAG can be used to inform the next direction of this group and future YPAGs. From our collaboration so far, we have found that the process of forming a YPAG requires extensive preparation. Researchers looking to establish future YPAGs in Ireland may benefit from availing of relevant webinars and workshops to upskill their expertise and training. One example includes the useful resources and guidance that is currently provided by an Irish group of researchers and patients from the PPI Ignite Network at Dublin City University [21]. The Lundy model is a useful aid that was developed for practitioners to effectively implement a child’s right to participate by focusing on the key concepts of space, voice, audience, and influence [39, 40]. Likewise, extensive work by Preston and colleagues have developed clear guidance and direction related to the ethics of involving children in research, the formation of YPAGs, and evaluation of PPI activities [19, 23, 41]. Additionally, flexibility regarding recruitment and communication strategies is necessary to tailor the involvement of young people based on their ever-changing lifestyles. Likewise, efforts to promote fun and opportunities for learning should be integral to the design and delivery of birth cohorts. Greater focus on these aspects may be critical in easing the challenge of maintaining recruitment and retention rates amongst young people involved in a longitudinal study.

Collaborations with YPAGs have proven successful and continue to enrich paediatric health research impact and relevance worldwide [3]. The International Children’s Advisory Network (iCAN) in 2015 is an international network of YPAGs within the ages of 8–23 years, to increase voice and impact on paediatric medicine and research [3, 22, 42]. Young people are involved in a range of projects including research consultations, revision of documents, and providing recommendations to research protocols [43]. One case of an effective output from a YPAG collaboration is the development of informed consent forms for paediatric clinical trials by the European Network for Paediatric Research, through work with the European Young Person’s Advisory Group Network [3]. This is a growing network of YPAGs aged 8 to 19-years who are involved in a variety of projects throughout the research process [3, 44]. Similarly, the Generation-R Alliance is a national network of YPAGs made up of 17 groups across the UK who support the design and delivery of paediatric research [44]. Groups consist of 10–15 members each, all aged between 8 and 19-years [44]. An example of engagement with one group from Generation-R in 2018 led to adaptations in the study design and patient information resources for two Pfizer Ltd studies trialling a new treatment for atopic dermatitis [45]. Likewise, the YPAG at the National Children’s Cancer Service in Ireland includes 12–29 year-olds with a previous or current cancer diagnosis [46]. Their role involves providing their views on patient information sheets and consent forms and offering advice on the design of paediatric clinical trials [46]. The aforementioned examples provide insight into just a few of many important outputs that can be achieved from successful collaboration with children and young people in research.

Future plans include giving the ROLO YPAG an opportunity to assist the research team in reading and designing age-appropriate, child-friendly information leaflets and resources for our future study visits. To support the unique identity of our YPAG, members will be able to express their creativity by designing a dedicated group logo. As mentioned, to improve the delivery and effectiveness of PPI in our research we intend to incorporate greater opportunities for feedback from YPAG members after each future meeting. The researchers anticipate that children will become more comfortable as future meetings take place in-person without the need to prompt feedback and that they will guide the future direction of their role that reflects their interests. As the ROLO children approach adolescence, increased effort is required to retain YPAG members. Future consideration may be directed towards liaising with Irish organisations such as Gaisce that may reward young people for their involvement in Irish research. Effective communication of the relevant benefits of participation will also be necessary to maintain interest. Factors such as skill development and receiving a certificate of attendance will benefit young people as they begin seeking employment opportunities [29].

The benefits of being part of a YPAG for children, researchers, and research itself are well-recognised [4, 6, 9]. The establishment of the ROLO YPAG is a step-forward in enabling the ROLO children and their siblings voices and thoughts to be translated into research questions of importance. As the group further develops, our aim is to provide the children with opportunities to participate in study decision making and planning, thus allowing them to feel valued through their active contribution [3, 43]. Including the older siblings in the ROLO YPAG allows children to collaborate and network with peers of different ages which has been demonstrated to contribute to increased confidence and self-esteem [3]. Children can develop a range of transferable skills in communication, research, and leadership, while learning new information about health research that is typically not provided in school.

For researchers, involving the views and opinions of young people in the ROLO study will provide new research perspectives and a greater understanding of this cohort [43]. The ROLO YPAG may contribute to improved study design and conduct as children continue to guide the acceptability and feasibility of our research. Engaging with a YPAG may also potentially increase recruitment and retention rates in the ROLO study, which is a critical aspect of maintaining a longitudinal birth cohort [3, 25]. Based on high quality evidence, it is expected that the dissemination of future results from the ROLO study may achieve greater impact and applicability through work with a YPAG [4]. On a wider context, the benefits of collaboration between children and researchers from the ROLO study may extend further across society. Heeding the voices of young people may lead to reformed public health policies that are more reflective of children’s health-related priorities and concerns [3].

The strengths of our YPAG include that the participants have a diverse age range. Older siblings of the ROLO children were included in this YPAG to provide support to their younger sibling and an external perspective that may reduce potential bias. Several forms of communication by email, phone, and social media were used to contact parents for the recruitment of YPAG members. The research team tried to facilitate as many children as possible, by hosting meetings during school term breaks and at times that best suited children. Meetings were recorded and transcribed verbatim for accuracy. Investigator’s triangulation was included in the theme analysis to reduce personal influence on the interpretation and enhance the study credibility.

There are several limitations worth considering. This YPAG is in its early stages, with a small number of members. While every effort was made to increase numbers, due to family, work, and school commitments this presented as a challenge. As the YPAG meetings have been held virtually so far due to the COVID-19 pandemic, the online platform has challenged the ability to build strong rapports with the children and to create a relaxed environment. The interpretation of themes may be limited by the inability to observe non-verbal communication cues naturally present in a face-to-face setting. As these are important aspects in the establishment of an effective YPAG, the research team intends to hold future meetings in-person. While several efforts were implemented to reach less-engaged participants, authors acknowledge that the YPAG is made up of highly engaged families. Recent research by Killeen et al. [47] reported factors such as personal interest as determinants of engagement in health-related research activities. It is also important to consider that the input of younger siblings may have been overshadowed by their older siblings, and future in-person meetings may enable siblings to be separated to provide participants with an equal opportunity to contribute in a comfortable setting. As children can be reserved in unfamiliar environments, the meetings were guided by topics which potentially limited opportunities for open discussion. This age group can also be vulnerable to concerns regarding self-image amongst peers which possibly hindered their contribution. Our findings and insights are limited to the themes that were identified from the meetings thus far; however, future research may explore key topics beyond those discussed in this report.

## Conclusion

Our experience so far of collaborating with the ROLO YPAG indicate that efforts to promote fun and opportunities for learning should be integral to the design and delivery of birth cohorts. Greater focus on effective recruitment and communication strategies may also ease the challenge of maintaining retention rates amongst young people involved in a longitudinal study. As our YPAG continues to develop, the incorporation of the views and opinions of our group will enrich the ROLO agenda and broaden future research pursuits in this birth cohort.

## Supplementary Information

**Additional file 1**. ROLO young person’s advisory group information.

## Data Availability

The datasets used and/or analysed during the current study are available from the corresponding author on reasonable request.

## Abbreviations

ROLO: Randomised cOntrol trial of a LOw glycaemic index diet in pregnancy to prevent macrosomia
PPI: Public and patient involvement
YPAG: Young Person’s Advisory Group
