# Supplementary material for: Including the child’s voice in research from a longitudinal birth cohort: insights from the ROLO young person’s advisory group
Source: Res Involv Engagem. 2023 Feb 9;9:2. doi: 10.1186/s40900-023-00411-y (PMC9910271; doi:10.1186/s40900-023-00411-y)

ROLO YOUNG PERSONS ADVISORY GROUP

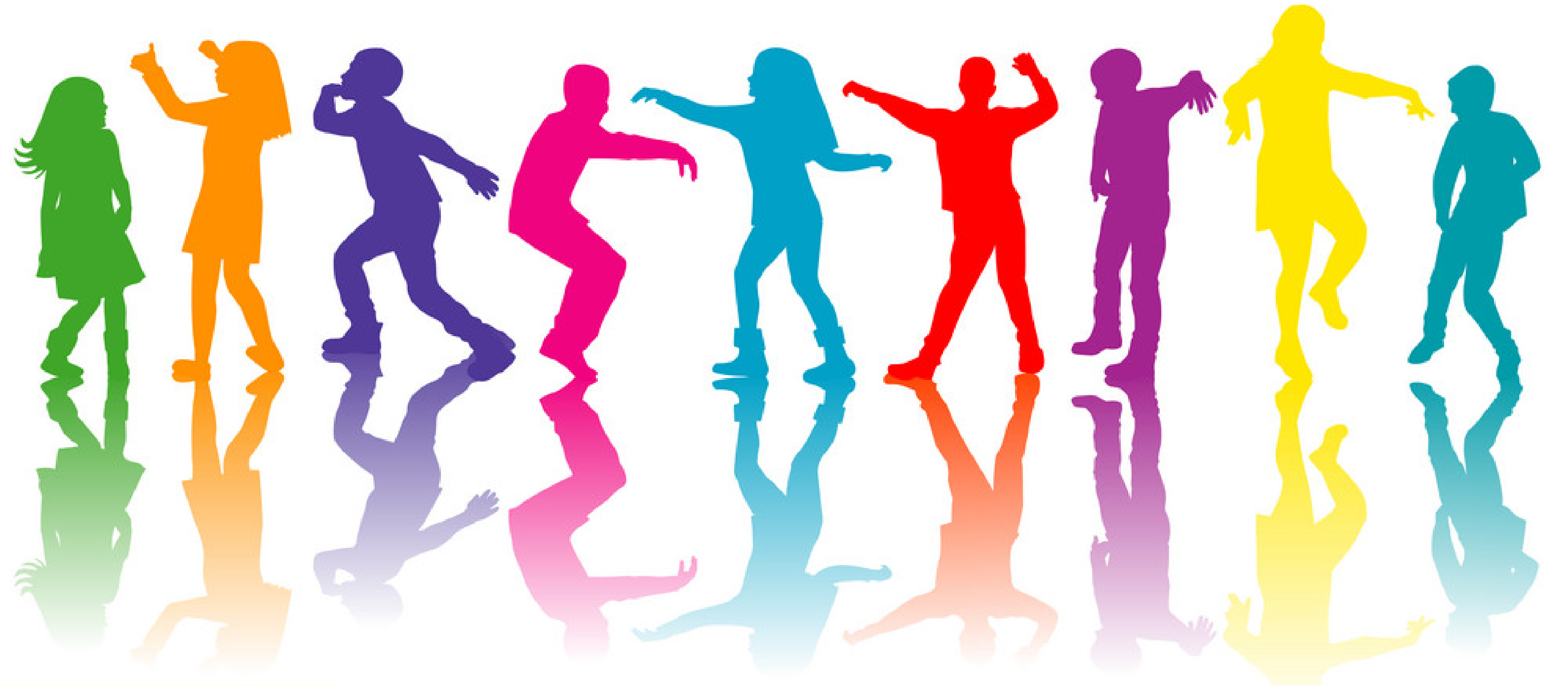

Hi everyone!

Thank you for your interest in the ROLO Young Persons Advisory Group! This group is being set up so that we can involve the ROLO kids in the research we are doing. In the past few years, the importance of involving research participants in planning and rolling out research has been recognised by the scientific community.

We have already established the ROLO Parents Advisory group but we would really value the kids' opinions as well. We would like to find out what ROLO young people think about our research and research in general and find out if they have any bright ideas about relevant research topics that we have not thought about!

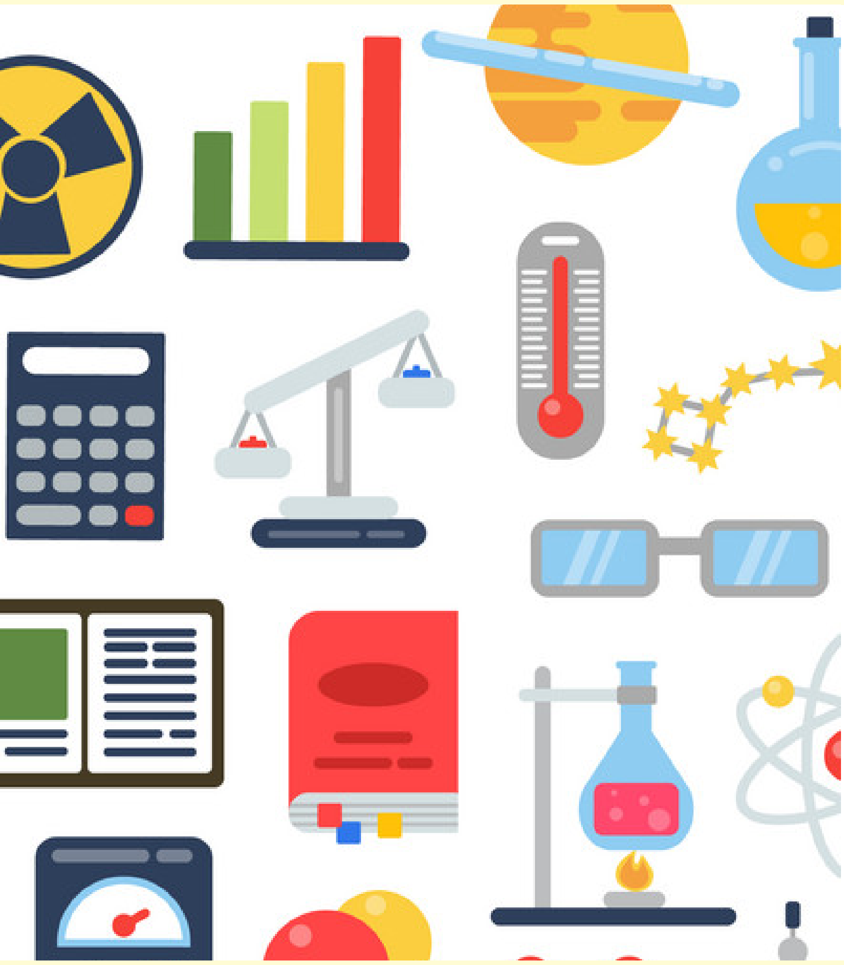

What does the Young Persons Advisory Group involve?

The Young Persons Advisory Group would involve having 2 meetings per year. For the moment, our meetings will take place via zoom and will last approximately 1- 1½ hours. In the future workshops or face-to-face focus groups may take place. The meetings will be very relaxed and hopefully interesting and fun for everyone!

Some of the topics we would like to talk to the ROLO kids and their older brother or sister about are:

- What they think is important for us to research in their age group and what is the best way to do this
- If they have already attended a ROLO study day we would love to hear about what their favourite part of the day was and if there is anything they would change about it to make it better
- How interested they are in science. We will spend some time explaining some of the scientific reasons behind why we do certain things on the ROLO study days and share some interesting scientific facts with you

We have also sent a short survey for Mum or Dad to complete. This is just to help us plan the meeting times/dates etc. Before the meeting we will send an outline of what we will cover.

We are really looking forward to meeting you. If you have any questions feel free to email us at: rolostudy@gmail.com or phone 089-2399498

Best wishes
Anna & Marie

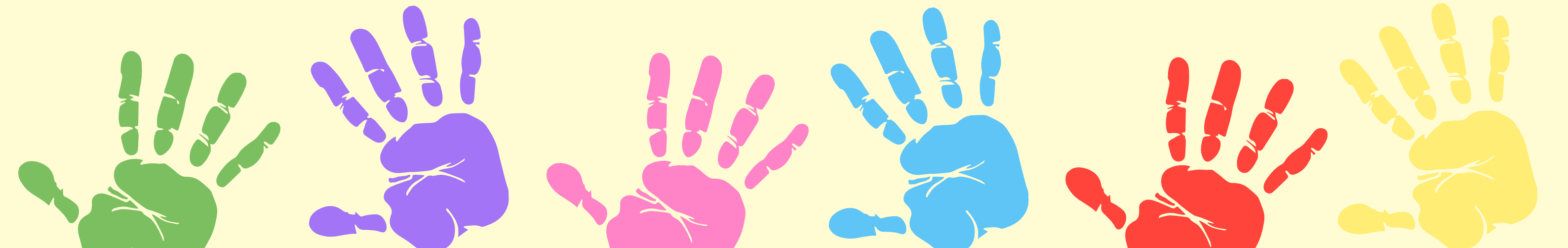

Supplement: Supplementary file 1 — Additional file 1. ROLO young person’s advisory group information. [file 40900_2023_411_MOESM1_ESM.pdf]
